# Influence of Retrogression and Re-Aging Parameters on the Microstructure and Hardness of Jet-Formed 7050 Alloy

**DOI:** 10.3390/ma18051063

**Published:** 2025-02-27

**Authors:** Lei Zhang, Shuohao Xing, Hongchao Zhai, Huiying Hou, Zhijie Wang, Sha Liu

**Affiliations:** 1 State Key Lab of Metastable Materials Science & Technology, Hebei Key Lab for Optimizing Metal Product Technology and Performance, College of Materials Science & Engineering, Yanshan University, Qinhuangdao 066004, China; zzhanglei0228@163.com (L.Z.); 18830896737@163.com (S.X.); 15133787552@163.com (H.Z.); houhuiying0310@163.com (H.H.); 2 School of Mechanical Engineering, Yanshan University, Qinhuangdao 066004, China

**Keywords:** 7050 alloy, regression and re-aging treatment, η’ (MgZn_2_)

## Abstract

In this paper, regression and re-aging treatment (RRA) was performed on the 7050 alloy, and the evolution in the microstructure of the 7050 alloy was observed by metallography and SEM. The effect of regression and re-aging treatment on the hardness of the alloy was investigated; the maximum hardness was 84.576 HRB at 180 °C/30 min. It was found that with the increase in regression treatment time, the size of the precipitates on the grain boundaries gradually increased, while the number of the precipitates inside the grain decreased accordingly. In the course of the experiment, the η’ (MgZn_2_) transforms into the η (MgZn_2_). As a result, the hardness of the alloy showed a decreasing trend. Meanwhile, the precipitates on the grain boundaries gradually increased in size, while the number of precipitates within the grain boundaries correspondingly decreased. These results reveal the influence of the re-aging treatment on the microstructure and mechanical properties of 7050 aluminum alloy, providing an important experimental basis for further optimizing the performance of the alloy.

## 1. Introduction

Al–Zn–Mg–Cu alloys are widely used in aerospace, transportation, and building structures due to their excellent mechanical properties, such as high specific strength, high toughness, and excellent machining performance and weldability, as well as corrosion resistance [1]. Currently, the development of Al–Zn–Mg–Cu alloys mainly focuses on the following two aspects: first, microalloying, by adding trace amounts of rare earth elements or other alloying elements to improve the microstructure and mechanical properties of the alloys. This method of adding trace elements is commonly found in aluminum–magnesium alloys [2,3,4]. Liu et al. investigated the effect of Sc and Zr microalloying on the microstructure and mechanical properties of 7xxx aluminum alloys with high Cu content during casting [5], deformation, and heat treatment. The addition of Sc and Zr not only refines the grains but also improves the hardness and yield strength of 7xxx alloys through the enhancement of the grain boundaries and the precipitate of Al_3_(Sc, Zr) by the addition of small amounts of Sc and Zr. The 7xxx alloy with 0.25 wt.% Sc showed the best mechanical properties among the prepared alloys. Wu et al. found that the addition of the trace element erbium (Er) can effectively improve the overall performance of aluminum alloys [6]. The addition of the trace element Er to aluminum alloys can form nano-sized Al_3_Er precipitates, and these thermally stable nanoscale precipitates can significantly refine the grain size, delay recrystallization, and improve mechanical properties and corrosion resistance. Hao et al. investigated the effect of the addition of the rare earth element Nd on the microstructure and mechanical properties of Al–Zn–Mg–Cu–Zr alloys [7], and found that the addition of Nd significantly refined the grain size and the dendrite size of the alloys, and effectively suppressed the recrystallization behavior during hot extrusion and solid solution treatment. The Al_3_Nd precipitates were in the vicinity of dislocations and hindered the movement of the dislocations, which led to an improvement in the mechanical properties of the alloys. The alloy with Nd could maintain a high hardness value at high temperature for a long time, and the tensile strength of the alloy with 0.26 wt.% Nd reached 396.2 MPa at 120 °C, which indicates that the rare earth element Nd can improve the high-temperature mechanical properties of the alloy. Deng et al. systematically investigated the influence of three elements, Sc, Zr, and Ag, on the microstructure and corrosion behavior of 2xxx aluminum alloy by micro-alloying [8]. The dendrite spacing of the micro-alloyed alloys was reduced, and the width of the precipitation-free zones (PFZs) of the alloys was changed. As a result, the corrosion resistance of the alloys was significantly influenced by micro-alloying.

The other is to modulate the alloy microstructure through different heat treatments and other means to obtain better overall performance. Some studies have shown that 7xxx series aluminum alloys can be strengthened by heat treatment and can reach 490.82 MPa [9,10,11,12,13]. Heat treatment of 7xxx aluminum alloys consists of solid solution, quenching, and stretching (to remove residual stresses), followed by several aging steps [14]. Qi et al. conducted solid solution heat treatments on 7050 aluminum alloy at different temperatures from 440 °C to 470 °C and kept for different time between 0.5 h and 8 h [15]. The grain size was enlarged significantly with increasing time and temperature, which resulted in an obvious decrease in the microhardness due to the dissolution of precipitates. Liu et al. investigated the mechanical properties and corrosion behavior of 7A46 aluminum alloy under peak aging (T6), double aging (DA) [16], and pre-aging (PA) followed by double aging (PA-DA). The hardness and strength of the 7A46 alloys were in the order of T6 > PA-DA > DA, and the corrosion resistance was in the order of PA-DA > DA > T6. The small and dispersedly distributed matrix precipitates (MPs) led to the high hardness and strength of T6 alloy, and the corrosion resistance of the alloy was enhanced. Liu et al. investigated the relationship between microstructure, mechanical properties, and corrosion behavior of sprayed 7055 aluminum alloy during peak aging (T6) [17], double aging (DA), and regression re-aging (RRA). The hardness test, tensile test, and impregnation test detailed the microstructural observation, and kinetic potential polarization was performed. The results showed that the corrosion resistance was in the order: DA >RRA > T6. Guo et al. investigated the effect of solid solution treatment on the microstructure and mechanical properties of the newly developed 7xxxaluminum alloy [18]. After solid solution treatment at 470 °C for 4 h and aged at 120 °C for 24 h, an electronic conductivity and peak aging hardness of 30.8% IACS (International Annealing Copper Standard) and 204 HV, as well as ultimate tensile and yield strengths of 661 MPa and 588 MPa, were reached, respectively. Among aging treatments, regression and re-aging (RRA) is more widely used in industry due to the higher overall performance. RRA consists of a three-step heat treatment process consisting of pre-aging, regression treatment, and re-aging, which improves the corrosion resistance of 7xxx aluminum alloys while maintaining their high strength. The pre-aging step is carried out in the peak aging condition to obtain uniform and fine precipitates. The regression treatment is conducted at higher temperatures to dissolve some of the precipitates. The re-aging treatment is conducted at lower temperatures, during which the GP zones are partially dissolved and the η′s are re-nucleated and will grow and even transform into η [19]. Pan et al. investigated the effect of RRA on the microstructure and fatigue behavior of 7050 aluminum alloy [20]. It was found that RRA treatment could obtain the same strength as the T6 state and improve the fatigue crack extension resistance. M.S. Nandana et al. performed RRA treatment on 7xxx aluminum alloy to study the effect of microstructural evolution on hardness [21]. The microhardness was 203 HV after 30 min of regression, and the RRA treatment resulted in the appearance of coarsened and discontinuous precipitates along grain boundaries, which improved the resistance to stress corrosion cracking (SCC). Zhang et al. investigated the effects of different RRA parameters on the microstructure, strength, and SCC resistance of aluminum alloys with high Zn content [22].

Jet forming, as one of the rapid solidification ways to produce aluminum alloy, has various advantages such as fine grain size, uniform microstructure, and the ability to inhibit macroscopic segregation. A large number of experiments have been performed by researchers, but there are very few reports on the optimum regression parameters of the jet-formed Al–Zn–Mg alloy. In this paper, jet-formed 7050 alloy was taken as an example, and then, through controlling the parameters of regression time and regression temperature, their effects on microstructure and mechanical properties were investigated.

## 2. Materials and Methods

### 2.1. Heat Treatment of Materials and Metallographic Observation

The material used for the experiments was a jet-formed 7050 alloy provided by CITIC Dicastal (Qinhuangdao, China), and its composition is listed in Table 1.

The alloy was held at 500 °C for 6 h to obtain a supersaturated α-Al solid solution, followed by pre-aging treatment at 120 °C for 24 h. Then, regression treatments were carried out by holding at different temperatures (180 °C and 190 °C) for different times (30 min, 60 min, and 90 min). Finally, the re-aging treatment was carried out by holding at 120 °C for 24 h. After each treatment, natural cooling was used to cool the alloy.

After re-aging treatment, the sample was ground and polished, and then rinsed with alcohol. The etchant for preparing the metallographic specimen was Keller’s reagent (HF:HCl:HNO_3_:H_2_O = 2:3:5:190), and the etching time was 7.5 s. After the corrosion process was completed, the surface was immediately cleaned with water, and then washed with alcohol. The purpose of corrosion is to make the grain boundaries of the polished side clearer. Then, the microstructure of the alloys was observed by an Axio Observer 3M metallurgical microscope, and the image magnification was 5 times, 10 times, 20 times, and 50 times.

For the same material, we performed peak aging (PA) at 120 °C/24 h as a control experiment for regression re-aging (RRA).

### 2.2. SEM Observation

After metallographic observation, the samples were then observed by Hitachi S-3400N Field Emission Scanning Electron Microscope (SEM, manufactured by Hitachi, Tokyo, Japan), The SEM equipment has a new 5-segment highly sensitive semiconductor backscatter probe, which can meet the low acceleration voltage and low vacuum observation of samples. The composition of the precipitated phases was analyzed by the implemented energy spectrometry (EDS). For EDS tests, the acceleration voltage was 15 kV, the beam current was 10^−8^ A, and the detection angle is 40°.

The sample was fixed on the SEM sample table with conductive tape to ensure that the surface of the sample is smooth and the conductivity is good. The sample table is placed in the SEM sample chamber, the equipment is vacuumed, and parameters such as accelerating voltage, beam current and scanning speed are set according to the experimental requirements, and the appropriate probe and observation mode are selected. The size and distribution position of the precipitated phase were observed by means of secondary electron imaging combined with the backscattering mode according to the difference of atomic contrast. At the same time, Energy Dispersive X-ray Spectroscopy (EDS) was used to qualitatively analyze the components of the precipitated phase. According to the experimental needs, the focal length, magnification and other parameters can be adjusted to obtain a clearer image. Image processing and analysis: The collected image data is imported into the image processing software for noise reduction, enhancement and other processing, and the sample surface topography, particle size, distribution and other characteristics are analyzed.

### 2.3. XRD Analysis

After SEM observation, the samples were re-polished. Then, the phases within were analyzed by a D/MAX-2500/PC X-ray diffractometer(XRD, manufactured by Rigaku International Corporation, Tokyo, Japan). The target material was Cu Kα; the scanning angle 2θ was set to 30–120° with a step of 0.02°. The scan time step was 1 s. After the test was completed, the collected diffraction data was imported into Jade for phase composition and crystal structure analysis, and the value of the diffraction peak was obtained. Origin software(version 8.1) was used to draw, and qualitative analysis was performed on the composition of the precipitated phase.

### 2.4. Hardness Test

The polished samples were tested by using LC-200R Rockwell hardness tester (manufactured by FUTURE-TECH CORP, Kawasaki, Japan). The indenter was made of steel with a diameter of 1.588 mm, and the experimental load was 100 kgf. Five points were taken on the surface, and the mean and error were calculated. We also performed a hardness test on the peak-aging (PA) samples.

## 3. Results and Discussion

### 3.1. Metallographic Microstructure

**Figure 1:**
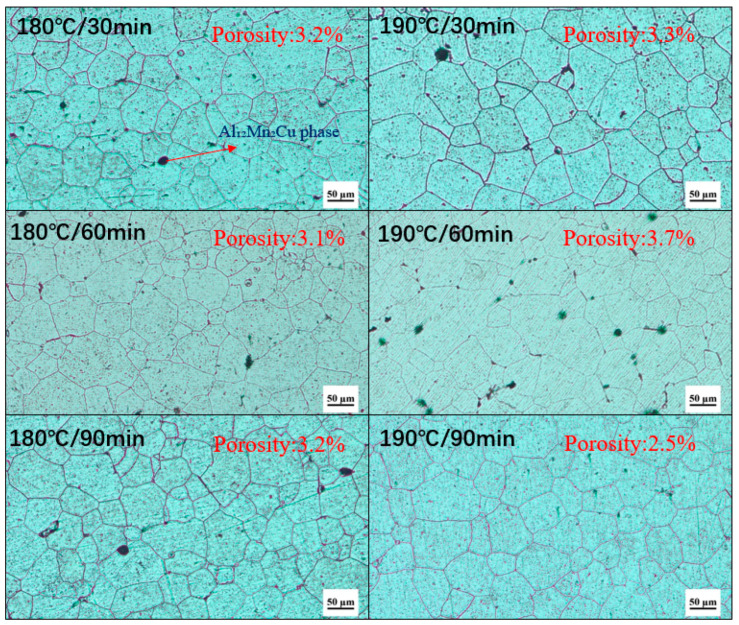
Metallographic microstructure of 7050 alloy with different heating temperatures and holding times.

Figure 1 shows the metallographic microstructure of the alloy after treatment with different parameters, and the size distribution of the grains. The irregular black dots in the figures are the presence of alloy impurities and porosity coexisting with grains. Impurities and pores in the alloy (irregular black spots in the figure) can affect the microstructure and coexist with the grains. The existence of these defects may affect the growth and distribution of grains [23]. It was found that with longer regression time at the same regression temperature, the number of small grains decreased, which means grains gradually grow. Similarly, as regression temperature rose, the number of small grains also decreased, as shown in Figure 1. The results show that with the prolongation of regression time, the number of small grains decreased and the grains gradually grew, and the increase in regression temperature would promote the grain growth, resulting in the decrease in the number of small grains [24]. Grain growth is a thermal activation process, and the increase in regression temperature and time will accelerate atomic diffusion and grain boundary migration, thus promoting grain growth [25].

**Figure 2:**
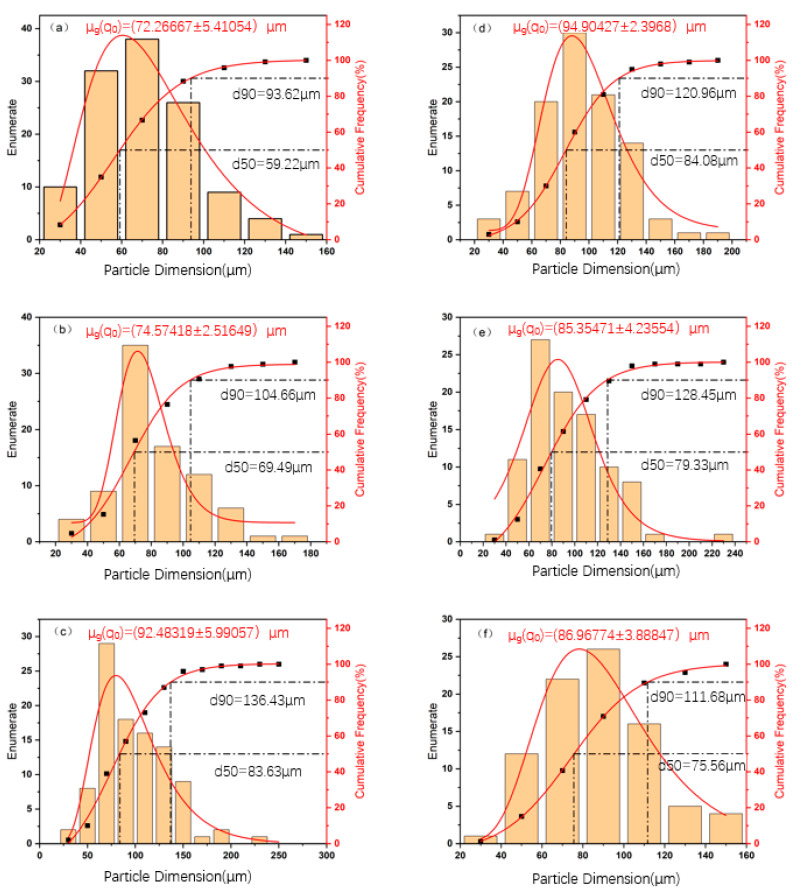
The size distribution of the grains (**a**) 180 °C/30 min; (**b**) 180 °C/60 min; (**c**) 180 °C/90 min; (**d**) 190 °C/30 min; (**e**) 190 °C/60 min; (**f**) 190 °C/60 min.

In Figure 2, μ_g_ (q_0_) indicates the geometric mean and standard deviation of the grain size. The value d90 indicates that 90% of the grains are smaller than this size, and d50 indicates that 50% of the grains are smaller than this size. When the temperature is constant, it can be seen that with a longer holding time, the number of large grains is increased. The results show that with the extension of regression time, the grain size distribution moves to the direction of larger size, and the number of large grains increases. This is consistent with the phenomenon observed in this paper, that 90% of the grain size (d90) gradually increases with the extension of holding time at a constant temperature [26]. In detail, when holding at 180 °C for 30 min, 60 min, and 90 min, 90% of the grains are smaller than 83.96 μm, 94.40 μm, and 97.38 μm, respectively, while, when holding at 190 °C for 30 min, 60 min, and 90 min, 90% of the grains are smaller than 111.68 μm, 120.96 μm, and 128.45 μm, respectively. When the temperature is constant, it can be seen that with a longer holding time, the number of large grains is increased. As can be seen from Figure 2, when the holding time is 30 min and 60 min, the proportion of large grains is more than that of small grains as the temperature increases, while, when the holding time is 90 min, the opposite trend is shown, and the average diameter of grains decreases. At longer holding times (such as 90 min), abnormal changes in grain size may occur, such as a decrease in average grain diameter. This is consistent with the phenomenon observed in this paper, that is, the proportion of large grains decreases with the increase in temperature at 90 min of holding [27].

### 3.2. SEM Image Analysis

**Figure 3:**
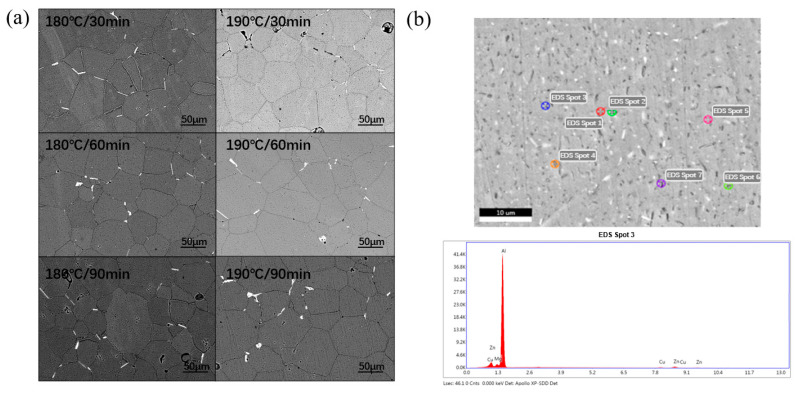
(**a**) Microstructure under scanning electron microscope; (**b**) EDS analysis.

Figure 3 shows the backscattered scanning images and EDS analysis. The uniformly distributed black dots within the grains are likely η precipitates. In general, a grain boundary (GB) with high intergranular energy is the preferred location for the nucleation and growth of aged precipitate. He et al. analyzed the coarser growth of intergranular precipitate according to the variation of PFZ width at the intergranular location [28]. It can be seen that the bright white regions in Figure 3a are distributed along the grain boundaries and dotted in the crystal. At the same time, there are also regions similar in shape to these white regions in the grain boundaries and in the crystal, but the color is black. Then, by observing the approximate number, shape, distribution, and other states, the second phase in the microstructure can be used as a reference. The long, bright areas distributed on the grain boundaries are the secondary phases consisting of Al, Mg, and Zn.

In terms of the quantity of the second phase, a longitudinal comparison is made first. We observe the regression temperature of 180 °C in Figure 3a, and it is obvious that the number of precipitates on the grain boundary is the least when holding for 30 min; the number of precipitates on the grain boundary is the middle when holding for 60 min; the number of precipitates on the grain boundary is the most when holding for 90 min; and the number of precipitates in the crystal is the most when holding for 30 min. The amount of heat preservation for 60 min is in the middle, and the amount of heat preservation for 90 min is the least. The reason is that the regression time gradually extends from 30 min to 90 min. The higher the degree of redissolution of precipitated phase in the crystal, the longer the precipitation time of the precipitated phase in the regression process, which corresponds to the continuous growth and coarsening of the precipitated phase in the grain boundary. With the extension of regression time, the intracrystalline precipitates redissolve and the grain boundary precipitates gradually become coarser. This is consistent with the phenomenon observed in this paper [29]. The same trend is also shown when the regression temperature is 190 °C. Then, we make a horizontal comparison to compare the influence brought by different regression temperatures under the same regression time. By comparing the samples with the same holding time, we find that the samples with higher regression temperature have a more obvious coarsening effect on the grain boundary and less second phase in the crystal. The density of the precipitated phase near the grain boundary is much lower than that far away from the grain boundary. The reason for this phenomenon is that the coarsening of the η phase consumes a lot of solute, and the content of the solute near the grain boundary is significantly reduced. Secondly, in the morphology of the second phase, it can be readily observed that the precipitated phase on the grain boundary is gradually coarsened from 30 min to 90 min, which is due to the increase in the regression time and the coarsening time of the precipitates on the grain boundary. The coarsening of precipitates near the grain boundaries consumes a large amount of solute, resulting in a significant reduction of solute content near the grain boundaries, and the density of precipitates near the grain boundaries is much lower than that away from the grain boundaries. This phenomenon is consistent with the low density of the precipitated phase near the grain boundary observed in this paper [30].

In order to further verify the precipitate type, the dots in Figure 3a were analyzed by EDS. Seven points were tested, and by comparison, the results of these seven points were similar, so we chose one point to analyze, of which the scanning result is shown in Figure 3b. GAI et al. also obtained similar second phase components by the EDX analysis method [31].

The obtained elemental contents are listed in Table 2. Based on Table 2, the Mg/Zn ratio is in the range of 1–1.4, which means the precipitated phase is MgZn_2_.

### 3.3. XRD Analysis Results

**Figure 4:**
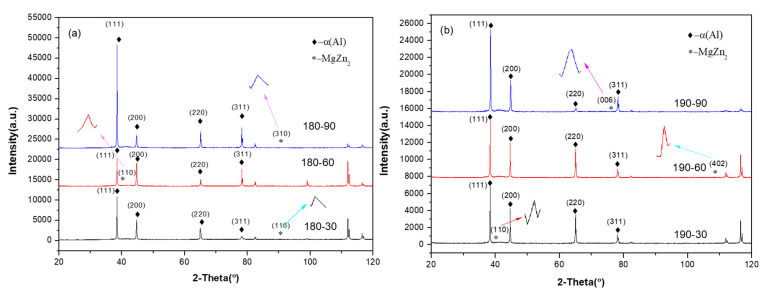
X-ray diffraction spectra recorded on the surfaces of heat-treated alloys for different parameters (**a**) 180 °C; (**b**) 190 °C.

The regression temperature and time have significant influences on the type and quantity of precipitated phase in aluminum alloy. XRD analysis can detect the precipitated phase of different crystal faces, such as (116), (110), (310), etc. [32]. Figure 4 shows the X-ray diffraction spectra of the specimens, where (a) is the spectrum of the specimens at three regression times at 180 °C, and (b) is the spectrum of the specimens at three regression times at 190 °C. From the XRD patterns, it can be seen that when the regression temperature is 180 °C, a small amount of precipitation phase is found at (116) when held for 30 min, (110) when held for 60 min and (310) when held for 90 min. When the regression temperature is 190 °C, a small amount of precipitation phase is found at (110) when held for 30 min, (402) when held for 60 min, and (006) when held for 90 min.

According to the analysis of the XRD pattern, α-Al exists mostly in the form of a solid solution, according to the theoretical analysis; in the state of regression, re-aging has been completed, the second phase should exist in the alloy as the η phase, and there may be a small part of the η’ phase; their composition is MgZn_2_, from the initial supersaturated solid solution state (SSSS) precipitated GP region. It is in a coherent state with the matrix, then gradually changes to the semi-coherent η’ phase, and finally, to the non-coherent η phase within the matrix. In the process of regression re-aging, the precipitated phase gradually changes from the GP region to the semi-coherent η’ phase and finally forms a non-coherent η phase (MgZn₂). This process is consistent with the results of XRD analysis in this paper; that is, the second phase is mainly the η phase, and there may be a small amount of the η’ phase [33].

The amount of the second phase is tiny, probably because the size of the second phase is too small to be detected. The sensitivity of XRD detection is affected by the size of the precipitated phase. When the size of the precipitated phase is too small, it may not be effectively detected by XRD. This explains the low number of second phases observed in this paper [34].

### 3.4. Rockwell Hardness

**Figure 5:**
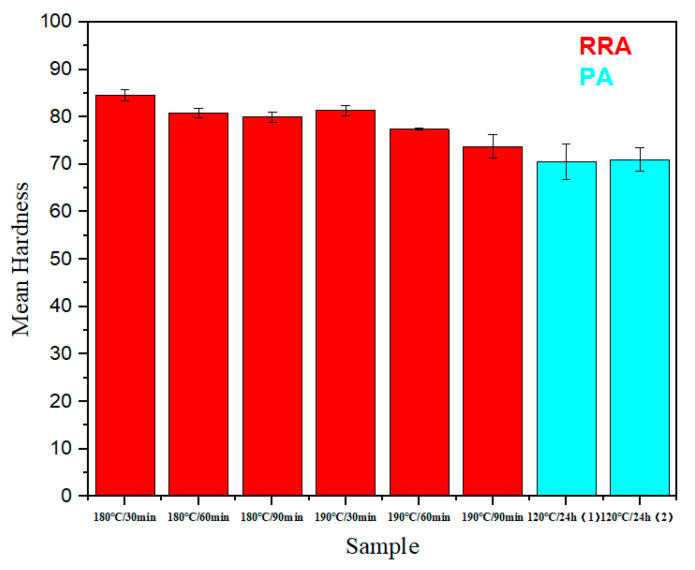
Average Rockwell hardness of each specimen.

A Rockwell hardness test was carried out on the samples after RRA and PA. Figure 5 shows the average hardness of the alloy after treatment with different parameters. It was revealed that when the regression temperature was constant, the Rockwell hardness of the alloy showed a decreasing trend with longer holding time. When the holding time was constant, the Rockwell hardness of the alloy decreased as the temperature rose. This is because in the regression process, as temperature rises or holding time is longer, the metastable η’ (MgZn_2_) phase will gradually transform into the stable η (MgZn_2_) phase. Since the main strengthening phase in the 7050 alloy is the metastable η’ (MgZn_2_) phase, it will lead to a reduction in hardness. This is consistent with the study of He et al., in which the hardness would decrease with the increase in insulation time [28].

In addition, as can be seen in Figure 5, the hardness after RRA is higher than that of PA, and the hardness is further improved.

### 3.5. Intergranular Corrosion and Stress Corrosion

Corrosion tests would provide additional insights into the performance of the alloy under different regression parameters. We have expanded the discussion in this section to include a thorough review of relevant literature on the corrosion behavior of 7xxx series aluminum alloys under similar heat treatment conditions. This provides a theoretical basis for the expected corrosion performance of our samples.

Yan et al. studied the stress corrosion behavior of 7050 aluminum alloy by mechanical tests and transmission electron microscopy. The results showed that the hydrogen content of the alloy had a great influence on its stress corrosion properties. This was due to the weakening zone produced by segregation of hydrogen in multi-adjacent grain boundaries [35]. Chen et al. studied, for the first time, the influence of precipitate size and state on the stress corrosion properties of 7050 aluminum alloy. The results showed that the 7050 aluminum alloy with fine precipitates and discontinuous copper-rich precipitates in the matrix had excellent stress-corrosion resistance, and different precipitates in the matrix led to electrochemical inhomogeneity and different corrosion sensitivities. In the stress corrosion process, discontinuous grain boundary precipitates can prevent the corrosion cavity from connecting into continuous cracks, while copper-rich grain boundary precipitates can delay hydrogen embrittlement, and it is also suggested that the precipitation state has a significant effect on passivation during the stress corrosion process. The fine nanoscale precipitation in the matrix is conducive to continuous passivation, and the copper-rich grain boundary precipitation is conducive to re-passivation [36].

Qiu et al. studied the effect of grain boundary precipitation on intergranular corrosion behavior through a stripping test and supplementary techniques. The results revealed the influence mechanism of grain boundary precipitates (GBPs) on intergranular corrosion behavior. Potential differences between GBPs and adjacent regions caused corrosion cavities to germinate along grain boundaries. In addition, the increase in active Mg and Zn contents in GBPs increased the potential difference and accelerated the germination of intergranular corrosion holes. The distribution of continuous precipitates in the grain boundary region contributed to the connection of initial corrosion holes, thus promoting the growth of intergranular corrosion cracks. Discontinuous GBP and precipitation-free zones (PFZ) hinder the diffusion and connection of intergranular corrosion cavities [37]. In short, after the regression re-aging treatment, whether for intergranular corrosion or stress corrosion, corrosion resistance was better than for other treatment methods.

## 4. Conclusions

In this paper, the effects of regression parameters on the microstructure and hardness of jet-formed 7050 aluminum alloy were investigated. This study provides a reference for the improvement of the properties of 7050 aluminum alloy and provides the support of heat treatment parameters for the development of 7xxx aluminum alloy in the future. The results show the following:

1. At the same regression temperature, with the extension of regression time, the number of small grains decreases, and the small grains grow gradually. Under the same regression time, the number of large grains increases significantly with the increase in regression temperature.
2. Through SEM analysis and EDS analysis, the precipitated phase is the metastable Mg–Zn phase. Through particle size analysis, it was found that the number of large-size grains increases with the increase in temperature and holding time.
3. XRD analysis confirmed that the precipitated phase was MgZn_2_.
4. With higher regression temperature or longer regression time, the hardness of the alloy decreases. The content of precipitates within the grain decreases. At 180 °C/30 min, the hardness number is the best; Rockwell hardness is 84.576 HRB.
5. Through the above experimental operation and result analysis, the best regression parameter in this experiment was 180 °C/30 min.

## Figures and Tables

**Table 1:** Composition of the alloy (wt.%).

Elements	Al	Zn	Cu	Mg	Zr	Others
content	90.5	6.14	1.70	1.36	0.17	0.13

**Table 2:** Content of elements in precipitated phase (wt.%).

Al	Zn	Mg	Cu	Precipitated Phase
89.28	4.28	4.53	1.9	MgZn_2_

## Data Availability

The original contributions presented in this study are included in the article and Supplementary Materials. Further inquiries can be directed to the corresponding authors.

## Supplementary Materials

The following supporting information can be downloaded at: https://www.mdpi.com/article/10.3390/ma18051063/s1, Table S1: Hardness value of RRA; Table S2: Hardness value of PA.
