# Supplementary material for: Influence of Retrogression and Re-Aging Parameters on the Microstructure and Hardness of Jet-Formed 7050 Alloy
Source: Materials (Basel). 2025 Feb 27;18(5):1063. doi: 10.3390/ma18051063 (PMC11901234; doi:10.3390/ma18051063)
Supplement: Supplementary file 1 [file materials-18-01063-s001.zip › materials-3489525-supplementary.pdf]

Table S1. Hardness value of RRA

Number	180℃ /30min	180℃ /60min	180℃ /90min	190℃ /30min	190℃ /60min	190℃ /90min
1	83.52	80	80.18	79.96	77.53	74.86
2	86.33	81.16	80.56	81.91	77.39	72.72
3	84.02	81.66	80.2	81.59	77.39	70.41
4	84.01	79.7	81.01	82.82	77.18	73.91
5	85	81.67	78.17	80.81	77.7	76.9
Mean value	84.576	80.838	80.024	81.418	77.438	73.76

Table S2. Hardness value of PA

Number	120℃/24h（1）	120℃/24h（2）
1	72.42	72.33
2	64.10	71.74
3	70.92	66.70
4	73.84	72.31
5	71.32	71.96
Mean value	70.52	71.008
